# A Role-Based Software Architecture to Support Mobile Service Computing in IoT Scenarios

**DOI:** 10.3390/s19214801

**Published:** 2019-11-05

**Authors:** Mariano Finochietto, Gabriel M. Eggly, Rodrigo Santos, Javier Orozco, Sergio F. Ochoa, Roc Meseguer

**Affiliations:** 1 GIDI, Department of Information Technology, Universidad Nacional de Mar del Plata, Mar del Plata 7600, Argentina; mariano.fino@fi.mdp.edu.ar; 2 SpinalCom, 91400 Orsay, France; 3 Department of Electrical and Computers, Universidad Nacional del Sur, ICIC-CONICET, Bahía Blanca 8000, Argentina; gmeggly@gmail.com (G.M.E.); jadorozco@gmail.com (J.O.); 4 Computer Science Department, Universidad de Chile, Santiago 8370456, Chile; sochoa@dcc.uchile.cl; 5 Department of Computer Architecture, Universitat Politècnica de Catalunya, 08034 Barcelona, Spain; meseguer@ac.upc.edu

**Keywords:** IoT-based systems, soft real-time interaction, communication model

## Abstract

The interaction among components of an IoT-based system usually requires using low latency or real time for message delivery, depending on the application needs and the quality of the communication links among the components. Moreover, in some cases, this interaction should consider the use of communication links with poor or uncertain Quality of Service (QoS). Research efforts in communication support for IoT scenarios have overlooked the challenge of providing real-time interaction support in unstable links, making these systems use dedicated networks that are expensive and usually limited in terms of physical coverage and robustness. This paper presents an alternative to address such a communication challenge, through the use of a model that allows soft real-time interaction among components of an IoT-based system. The behavior of the proposed model was validated using state machine theory, opening an opportunity to explore a whole new branch of smart distributed solutions and to extend the state-of-the-art and the-state-of-the-practice in this particular IoT study scenario.

## 1. Introduction

Mobile service computing (MSC) is a new paradigm that merges the service computing and mobile computing paradigms. Deng et al. [1] identify three macro-scenarios (or deployment patterns) to provide and consume software services: cloud-to-mobile (C2M), mobile-to-mobile (M2M), and a combination of the previous ones (hybrid). In the first scenario, the services are deployed in the cloud or in servers and consumed by mobile users. The cloud acts as an intermediary that stores and processes data and also provides services that allow mobile users both to update and consume such data. Examples of applications that use this deployment pattern are Waze and Foursquare. This pattern is widely used today since it has several benefits, e.g., its simplicity to structure applications, but it requires a stable communication link between the cloud and the mobile units.

In the second scenario (M2M), each mobile device performs point-to-point interactions with others devices without using mandatory interim units like servers or the cloud. In this scenario, the devices consume and provide data and services in an ad hoc manner, typically using opportunistic networks. Examples of applications that use the M2M deployment pattern are those that interact with digital assistants, like Amazon Echo or Google Home, where the communication between the devices is ad hoc and peer-to-peer. Provided that, in M2M scenarios, the communication support does not depend on having available communication infrastructure (e.g., 4G or Wi-Fi networks), the applications using this deployment pattern tend to be more robust in terms of ad hoc interaction capability. This pattern takes advantage of stable communication links, but it does not necessarily require them to provide or consume services. For that reason, M2M approaches are frequently used by applications that support emergency response processes or collaboration activities in areas without infrastructure-based communication.

Finally, hybrid scenarios are those in which parts of the system use C2M interactions and other parts use M2M settings. This heterogeneous scenario is the most representative of the Internet of Things (IoT) operating environments. For instance, in a home surveillance system, the sensors and actuators regularly use a M2M approach to interact with the control unit that detects unauthorized entry attempts. Typically, this control unit uses a C2M scenario to deliver alerts to police, security companies, and the inhabitants of that house.

Several challenges have been identified in implementing mobile service computing in hybrid environments, for instance, security, communication, efficiency, and context-awareness [1]. Particularly, for an ample variety of IoT-based applications, interaction support and data analysis in real time is required, since they usually involve monitoring, autonomous decision-making, and delivery of early notifications that represent time-constrained activities [2,3,4,5]. If the system does not react on time, its effectiveness is under risk. This requirement becomes evident in IoT systems that detect falls of older adults living alone or those that deliver early warning of natural hazards.

The real-time interaction support has been addressed by several research works, but the current proposals assume availability and stability of the link among the system components. Although that is fine for many applications domains, it is not for others where the communication support is uncertain, for instance, in natural hazard early-warning systems that usually involve several public and private networks to detect and notify extreme event to the population (e.g., floods, wildfires, landslides, tsunamis, and volcano eruptions). Provided that these actions should be done as quick as possible, soft real-time interaction support on a network with uncertain stability is required [6,7].

This article addresses the real-time interaction support for devices participating in a service-oriented IoT system that uses unstable communication links. This communication challenge has been poorly addressed in the literature, particularly when the service provider and consumer interact through a communication link with uncertain stability. In IoT environments, this type of interaction scheme is frequent due to several reasons, like the wide variety of network interfaces used to support these interactions or the number of autonomous solutions that coexist and interoperate in the same environment. After an extensive literature review, we have found no communication model able to support real-time interactions among components of an IoT scenario that considers unstable communication links.

The main contribution of this paper is the introduction of an MSC architecture, named Software Real-Time Interaction (SRTI), that involves the roles played by the different participating devices (publishers, subscribers, and brokers) and also a dynamic among roles to provide and consume these software services within time constraints. This architecture facilitates the inclusion of timestamps to handle soft real-time traffic, even under unstable networks conditions. A library extending the Mosca project [8] is presented to prove the concept, which also includes a graphical interface to define the aggregating and processing functions of the publishers. This proposal was evaluated using simulations, and the obtained results are highly promising.

The rest of the paper is organized as follows. Section 2 presents and discusses the previous works focusing the discussion on the capability to provide real-time QoS in the study scenario. Section 3 describes the structure and dynamics of the SRTI model at the architectural level; it also presents and justifies the main design decisions behind the model. Section 4 specifies the behavior of the model components, considering a publish–subscribe interaction scheme. Section 5 proposes modifications to the MQTT (Message Queue Telemetry Transport) protocol in order to translate the SRTI model into a messaging system for IoT-based application development. Section 6 presents three different experiments that illustrate the way in which the system works. Section 7 shows an application scenario based on precision agriculture, and Section 8 discusses the proposed architectural model considering the current literature in such a study domain. Finally, Section 9 summarizes the contributions of this proposal and specifies some lines of future work.

## 2. Related Work

As discussed before, most IoT solutions involve time constraints to gather and process information, to make decisions, and to deliver actions that system components must perform. When time restrictions are present, the system is said to be real-time if at least one of the tasks to be performed must be executed before a certain deadline. This constraint is difficult to comply with when the communication links are unstable or if they are subject to huge throughput variations due to traffic load.

Next, we present a review of the related work considering time constraints in the three aspects involved in this proposal: IoT structural models, IoT applications, and data processing technologies and models to support the applications.

### 2.1. IoT Structural Models

In their seminal paper, Deng et al. [1] present and discuss the opportunities and challenges to provide mobile service computing (MSC) in three scenarios, but particularly in those adhering to hybrid deployment patterns, which have a structure similar to IoT scenarios. Among these challenges, the authors raise the need to study the communication aspects that affect service provision and consumption, particularly when the communication link is unstable or uncertain, since it impacts the availability of these services. In such a paper, the authors present three interaction scenarios: C2M, M2M, and a mixed of them named hybrid. The need to keep the connection alive, even when some of the participating devices are on the move, is a common problem in these interaction settings since it determines the real capability of the system to support real-time interactions.

Most IoT interaction models are based on two interaction approaches. One of them involves a publish–subscribe mechanism in which some devices act as data providers by publishing specific topics in special nodes named brokers, while others act as consumers of those topics by subscribing to services provided by the brokers. This approach is implemented by protocols like Message Queuing Telemetry Transport (MQTT) [9]. The other approach is related to a more classical client–server structure in which certain nodes request data, while others respond to the request, using a very simple and lightweight protocol like the Constrained Application Protocol (CoAP) [10]. None of these strategies support real-time interactions.

The definition of architectures or infrastructures to support interactions in IoT scenarios follows the same line as the previous works. For instance, a scalable distributed architecture for IoT is reported in [11], where the authors propose a comprehensive communication model that does not contemplate time constraints. Similarly, in [12], the authors introduce a process calculus approach to formalize an IoT communication infrastructure. Although this proposal addresses several aspects of the message exchange, real-time considerations are not taken into account.

A cognitive machine-to-machine communication for IoT systems using a stack perspective is introduced in [13]; however, time is not included as a critical variable within the analysis of the proposal. In [14], the authors use the BIP (behavior, interaction, and priority) component framework [15] to model and reinforce the correctness of resource-constrained IoT applications. Similar to the previous works, time constraints are not considered as a critical value within the evaluation.

Following a different approach, Aziz [16] proposes a formal method for representing and analyzing an IoT communication infrastructure. The correctness of the communication model representation is evaluated using formal methods, and the implementation of the system uses a publish–subscribe paradigm analyzed based on the MQTT protocol [9]. Although in this proposal the time constraints are considered as a variable, the author does not provide a mechanism to validate the time requirements of the system.

In [17], the authors proposed an extension to MQTT for running on top of opportunistic networks or disrupted networks for IoT applications. Under this situation, the nodes use the store-and-forward mechanism as the transmission is only possible at the moment in which there is a connection between the nodes. The authors do not analyze the behavior of the system from a real-time point of view.

### 2.2. IoT-Based Applications

Several researchers highlight the need to extend our knowledge of wireless sensor networks and cloud computing, as instruments that support IoT software applications, and to understand the role played by big data, information sharing, and collaboration for IoT-based service provision [1,18]. Particularly, information sharing and collaboration on these infrastructures impose several challenges to the systems designers. Depending on the service to be provided, real-time interaction support can be required.

In [19], the authors present several IoT-aided robotics applications that operate under time constraints. However, these systems use dedicated or local area networks; therefore, its proposal to provide real-time interaction support among system components does not consider the potential instability of the communication links. In this case, delays in communication links are analyzed with traditional real-time instruments, as the network is dedicated to the application. A similar work is reported in [20], where the authors propose a low-cost wireless marshalling module for industrial environments, also based on dedicated networks.

In [21], the authors introduce the concept of the sensing cloud, where the Internet is used as the main communication network and the cloud represents the place where data can be stored and retrieved for its processing and use. Although this proposal presents a generic architecture and shows an implementation of it, there is no real-time consideration in the model; therefore, it cannot be used to support interaction in the studied scenario.

A similar proposal is described in [22], where the authors present a ubiquitous monitoring method to support IoT-based Emergency Medical Services. In this scenario, the information retrieved from the sensing devices is shared across the Internet, but the proposal does not consider real-time guarantees in the interaction process. For the same application domain, Koley and Ghosal propose an emergency communication and location tracking system that helps reduce damages in vehicular emergencies [23]. Although this system addresses real-time interactions, it does not formally consider the possibility to have unstable communication links among its components.

Precision farming (or agronomic) is also an area in which soft real-time communication is required. In [24,25], the authors describe the use of information technologies to improve the productivity of farms using decision support systems that are fed with data coming from sensors.

The introduction of new functionalities in long-term evolution (LTE) cellular technology promotes the development of new applications in the field of machine-to-machine communications. In this sense, in [26,27], the authors analyze the performance of a Long-Term Evolution Advanced (LTE-A) network for applications in Vehicle-to-Infrastructure (V2I) and Vehicle-to-Vehicle (V2V) communication. Moreover, they propose a mechanism to offload vehicles with low signal-to-interference-plus-noise ratio (SINR) to be served by other vehicles, which have much higher quality link.

In [28], the authors implemented a simulator called LTEV2Vsim, which allows managing vehicles mobility and performs communication packet allocation following different algorithms that are both network controlled and autonomous. The tool is interesting to represent IoT scenarios with high mobility but considers stable communication links among the nodes.

Concerning the way in which IoT devices connect to the Internet, it can be done in several manners. Many times, these devices are part of private networks that have access to open networks through a gateway. In these cases, there are several options at the link layer protocol. For instance, for 802.11, it is possible to use LoRa [29], Sigfox [30], and the one devised for vehicular networks like 802.11p [31]. Other times, the devices may be connected to the Internet using a 4G network. In the near future, there is an open discussion about the role that the next 5G protocol will have in relation to vehicular networks [32] and how they will interact with other devices [33]. The discussion is centered around the latency in the communication and the way in which messages are actually forwarded among the nodes participating in the network [28,34].

All these proposals are representative of many others that share similar strengths but, particularly, weaknesses to support real-time interactions in unstable networks. Therefore, a first question that is raised is how are the current systems addressing these interaction scenarios? The answer is simple: by relaxing one of these conditions. If real-time communication is mandatory, then the network should be stable (e.g., dedicated). In other cases, the current proposals cannot ensure a real-time QoS; therefore, no critical service should be provided through such an infrastructure. If we need to address both conditions simultaneously (as in many remote monitoring systems), a new communication model should be defined to support the interactions among the system components.

### 2.3. Real-Time Communication in IoT-Based Scenarios

After conducting an extensive survey on real-time data processing technologies and models to support IoT applications, Yasumoto et al. [35] found no protocol or implementation capable of providing real-time QoS when working with open Internet (or unstable networks). However, the literature reports several interesting works that can be used to support the definition or analysis of communication proposals in such a study domain. For instance, in [36,37], the authors introduce a temporal analysis of the CoAP [10] protocol, that allows to measure the latency or delay in the data transmission. This is useful to determine the performance of a particular protocol or link; however, it is not enough to guarantee real-time constraints or QoS. Related to CoAP, in [38], the authors analyze an extension to this protocol by introducing new real-time primitives that are validated through finite state machines.

On the other hand, Kolozali et al. [39] introduce an annotation procedure to deal with real-time data streams within IoT applications, but this research work does not indicate how the procedure contemplates associated deadlines and how these are verified with scheduling tests. The reported analysis is limited to low latency/delayed use of the network.

The Object Management Group (OMG) proposes the data distribution service (DDS) for real-time systems specification [40] that is implemented as a middleware based on Common Object Request Broker Architecture (CORBA). This proposal deals with message exchange among different components in the network. The interactions on DDS adhere to a publisher/subscriber mechanism, and the interaction model contemplates different QoS, including real-time [41]. In order to do that, DDS introduces various attributes like deadline, latency_budget, and periodicity. Moreover, it counts on several implementations like RTI Connext DDS (proprietary) https://www.rti.com/products/dds and OpenDDS (open source) http://opendds.org. Although this proposal is highly comprehensive, well-specified, and validated, the real-time support that it provides requires a communication link that is stable over time. Therefore, only the concepts behind this proposal formulation can be reused if we want to provide real-time QoS in the study scenario.

In [6], the authors proposed the use of Unmanned Aerial Vehicles (UAVs) to provide connectivity in disaster-relief scenarios establishing a flying ad hoc network. There are two main aspects considered in that proposal: the real-time scheduling of messages and the nodes deployment. Although the communication is performed using mobile devices, the network is dedicated and not open to the wide Internet. In [7], a real-time analysis for disrupted networks is provided when mules are used to link disconnected nodes. The schedulability analysis in this case contemplates not only the periodicity of the messages and the available bandwidth, but also the visiting order of the mule and the actual speed with which it moves around. Like in the previous case, the network is not considered to be an open Internet application, but a special ad hoc one created to support the action of firefighters during wildfires.

In order to address the stated challenge, the next two sections present the proposed interaction model design and its implementation, respectively. This model, named SRTI (Simple Real-Time Interaction), was designed to support soft real-time interactions in IoT scenarios that involve communication links with uncertain stability.

## 3. Proposed Architecture

Any interaction model proposed to deal with the stated challenge must define two basic aspects of the solution: the structure of the interaction scenario and the behavior of the interaction model. The first one establishes the components that will be present in the IoT scenario and the role played by those components. The second aspect describes the dynamics of the interaction model and the way in which the interaction services are provided considering the preestablished restrictions. Next, we present the design of these two aspects in the SRTI model and discuss the rationale behind the main design decisions.

### 3.1. Structure of the Interaction Scenario

**Figure 1:**
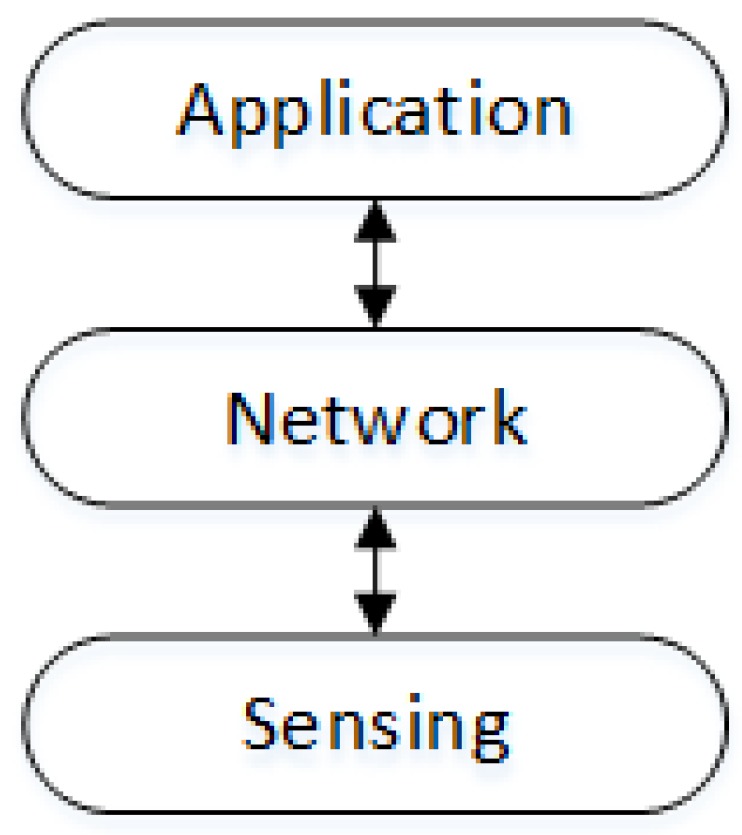
Layered structure of an Internet-of-Things (IoT) interaction scenario.

There are several alternatives for structuring the IoT interaction scenario; however, most of them implement a three-tier architecture that involves the sensing, network, and application layers [42] (Figure 1). This architecture is devised to coordinate the functionality of the nodes in order to obtain an integral behavior of the system, through the integration and coordination of its parts. This structure is not related to the traditional five-layer Internet model; in fact, any IoT system should count with transport, network, and link layer-nested protocols to operate in the open Internet network.

The lower layer involves the collection of physical devices that participate in the system, for instance, sensors and actuators that interact with the physical environment or smart objects acting as interaction intermediary or temporal data repository. The network layer is responsible for connecting all components in order to allow interactions and to coordinate the individual behavior of the devices to generate a collective behavior of the system (or subsystem). Therefore, this layer transports the information among components of the system and it implements mechanisms to address several communication aspects like modulation, medium access control, routing, transport, and flux control.

Finally, the application layer provides the system services to end-users; it includes human users, other systems, and also components of its own system (e.g., actuators). The complex behaviors of the system, for instance, making contextualized decisions, is usually implemented at this level.

In this architectural model, a node may act on the three layers, as it may be a sensor unit that transmits the collected data after processing and transforming it in information. Some nodes may collect data and information and then organize it following certain criteria or filters to forward it to other nodes. Finally, some nodes make decisions based on the information they receive.

**Figure 2:**
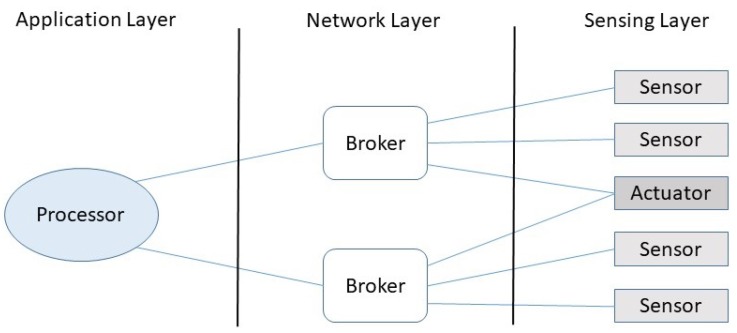
Hierarchy of the interactions among nodes.

The SRTI model adheres to such a structure considering three type of nodes: terminals, brokers, and processors. There are different kinds of nodes in the system, and some of them should implement different services at each layer. The terminals are represented by the sensors and actuators present in the system. Clearly, these are part of the sensing layer and provide simple services to brokers (Figure 2). A terminal can be linked to more than a broker, and such a link is loosely coupled. In case of actuators linked to more than a broker, every change in the device’s status is propagated to all involved brokers, following a change-propagation mechanism similar to the one proposed to the model-view-controller pattern [43].

The brokers are interim nodes that interact with a subset of related terminals in order to provide some basic intelligence at the middle level. Therefore, they implement several services based on the terminals linked to them. For instance, a broker can be designed to interact with all sensors and actuators related to the security aspect of a smart home, while other brokers can address other aspects of the system, like energy consumption, resource provision, or environmental wellness. In this scenario, the brokers act like a switch, connecting sensors or actuators with consumers in the application layer.

Finally, the processor nodes usually increase the intelligence of the system using the services provided by the brokers. Like the previous case, a processor can be linked to multiples brokers and vice versa. Processors are implemented in the application layer. The management of events in this layer usually adheres to the publish–subscribe model [41,44]. Unlike client–server or request–response coupling models, publish–subscribe facilitates the inclusion and exclusion of devices and also the dynamic orchestration of services provided by the nodes, making the systems more flexible and easy to evolve [44,45].

Considering the implementation aspect, it is important to remark that the node types represent conceptual constructs; therefore, they can be hosted in the same device, particularly the brokers and the processors.

### 3.2. Intelligent Management of Topics in the Communication Scenario

The IoT model described in the previous section is concerned with the functionality of the nodes and not with how the information is exchanged among the nodes in the network. Provided that we consider open IoT applications executing on the open Internet when the devices should use links provided by Internet Service Providers (ISPs), it is necessary to describe the interaction between the nodes in terms of the traditional network layers. As mentioned before, the IoT network layer comprises all the communication aspects related to the information exchange, and these are represented by the physical, link, network, and transport layers in the traditional Internet model.

Although IoT applications assume sensors and actuators are reachable through an open Internet in a flat access mode, it still has several practical restrictions like dynamic discovery of services, information providers, and routing. However, even if these restrictions are properly addressed, the network would require a huge bandwidth to avoid traffic overflow and congestion. The use of brokers in this scenario reduces the traffic, as they collect information from publishers and send it to subscribers on demand.

The brokers have an intelligent real-time agent (IRTA) associated in the application layer. This agent is subscribed to all the published topics in the broker on one hand, while it receives also all the subscriptions to the broker. A published data is filtered by the agent according to a set of functions that may be configured by the end user of the system. Each publication has metadata describing attributes like its publication period, precision, source, and reliability.

On the other side, subscriptions require data from certain sources, with an expected periodicity, precision, and reliability. In the case of a direct match between the subscription and the publication, the IRTA connects both. If there is not a direct match, the IRTA may work with the available publications to satisfy the subscriber with a new one. To do this, it may aggregate data from multiple sources to generate a desired sampling frequency not available within the publisher sources or fusion raw data in order to generate new information.

**Figure 3:**
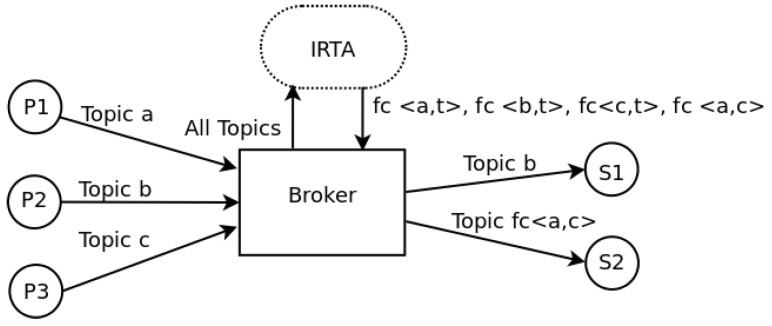
Structure of a broker.

The IRTA is in charge of checking that data is on time and of implementing scheduling policies in case of conflicts. The functions of the IRTA are not related to a server or the cloud. Instead, these functions only preprocess data to conform subscriptions and to validate it from a real-time perspective. If there is a subscription that requests certain periodicity for a particular data or requires a certain latency in the network to take a value as good, the IRTA should evaluate if publishers have data that meet these requirements. Using the last measured delays in the network, the IRTA can determine if it is possible to satisfy the minimum latency. Subscription is rejected if both things are not guaranteed. Figure 3 presents a diagram that shows this structure.

#### 3.2.1. Behavior of the IRTA

Typically, when the broker receives a subscription request that does not have a direct match in the publications, it calls the IRTA to analyze if the answer can be built using the data stored in the local repository. If it does, the IRTA generates a new topic matching the subscriber request under certain real-time restrictions. To do this, the user may implement a set of functions to work on the metadata associated to the publications.

On the other hand, when the IRTA is not capable of building the answer with the local information, a second negotiation stage is started and the requested topic is searched among different brokers participating in the system. In this second round, neighbor brokers share information to build the requested answer.

#### 3.2.2. Application Example

In order to illustrate this point, let us suppose there is a temperature sensor that provide the actual temperature read, together with additional information. This additional information includes the units in which the data is provided (i.e., Celsius, Fahrenheit, or Kelvin), the actual location of the sensor in a qualitative and precise way (e.g., Buenos Aires, latitude, and longitude), the sampling frequency or period with which the temperature is read, its precision (8,10,12 or 16 bits), and a last field that weighs the reliability of the sensor from the source. The metadata associated with this publisher provides the IRTA with the necessary information to aggregate or combine it with other data and, in this way, to obtain new information useful for the system or the end-users. Table 1 presents the data structure of the answer provided by the sensor.

Changing the units of the answers provided by the sensor is a simple operation, and it can be implemented with just a few computations. Functions associated to unit changes are notated U(a,b), where *a* is the unit used by the sensor and *b* is the desired unit. The frequency is more difficult to adjust. When a subscription requires certain sampling frequency, there are basically two scenarios to consider: (1) the publisher sampling rate is below the demanded one; or (2) it is equal or higher. In the first case, the only way to accept the subscription is to obtain more data from other sources. In the second case, there are two alternatives: either the subscriber accepts a higher data rate update or the IRTA builds a regression function (probably first order) to interpolate values matching the demanded frequency. However, to do this, deadlines should be large enough to admit the necessary latency to build the “computed” data, for instance, using the least squares methodology. The set of values used to do the regression can be dynamically updated.

In the case that a higher sampling frequency is required, the IRTA should check if it is possible to combine different publishers. In order to do that, the location, precision, and reliability of the sensors should be similar to produce a new publication with the necessary quality. In the basic case, let us suppose there are two temperature sensors in Buenos Aires with similar parameters and even the same period. Both produce data every 30 min, but the first one does it at o’clock and past 30 min while the second does it at 15 and 45 min. Combining both readings, the IRTA can provide the temperature with a 15-min period. The functions related to frequency adjustments are notated as F(a).

When a subscription requires data that is not present in the broker, the IRTA may ask the linked brokers to determine if they have a publisher satisfying the subscription. If there is such a broker, the IRTA may pass through the request to that broker, acting as a simple switch between the subscriber and the new pair broker/publisher. The function of searching the topic in another broker is notated as S(a,b), in which *a* is the original broker and *b* is the new broker.

The last functionality of the IRTA is related to providing information based on raw data. For this, it should be prepared with the appropriate functions, which may be as simple as providing the maximum or minimum values of a published topic or as complex as to compute a Fast Fourier Transform on a sequence of data to provide the spectrum of a topic. There are many options to be implemented according to the type of data processing that is required. In any case, these functions are notated as P(a), where *a* indicates the topic that is processed.

Using this architecture, the system can be seen as a hierarchical set of endpoints (i.e., sensors or actuators), intelligent real-time brokers, and subscribers. The brokers may have two functions as they can become publishers and subscribers of other brokers that allow them to get information not present within their inputs or outputs.

**Figure 4:**
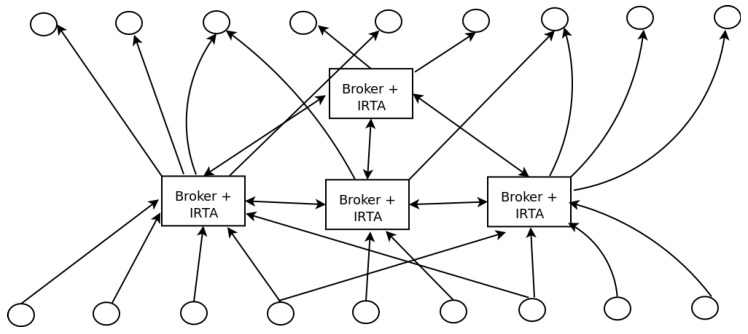
Structure of an interaction scenario.

Figure 4 illustrates an example of the general structure of the interaction scenario, considering an architecture with four interconnected brokers. For simplicity, publishers are in the bottom while subscribers are in the top of the figure. As shown in such a figure, several publishers share their topics with different brokers, and various subscribers receive topics from different brokers. In the middle, brokers share data as publishers and subscribers indistinctly based on the functionality required for each connection.

### 3.3. Real-Time Communication Model

A real-time task is defined as one that should produce correct results within a certain instant named deadline, and a real-time system is one in which there is at least one real-time task [46]. In an IoT scenario, these tasks represent threads that support the interaction among the system components, which are coordinated by one or more software applications. Therefore, a real-time task, $\tau_{i}$, is described as a sequence of jobs or instances.

The interval between these instances is named the minimum inter-arrival time or period, $P_{i}$. Each instance has a worst case execution time denoted $C_{i}$ and a relative deadline, $D_{i}$. Each instance has a release or activation time $a_{ij}$ and an absolute deadline defined as $d_{ij}=a_{ij}+D_{i}$.

There are several scheduling policies to order the execution of tasks, where the most used ones are Rate Monotonic Scheduling (RMS) and Earliest Deadline First (EDF). In RMS, task priorities are assigned in reverse order of *P*; therefore, the highest priority is assigned to the task with minimum *P*. In EDF, the priorities are dynamic and the highest priority is given to the task that has the earliest deadline. In this proposal, we adopt the earliest deadline first given it is optimal for single processors systems, as shown in [47].

In an IoT real-time interaction, the processing at both endpoints (i.e., the producer and consumer) and the delay in the network have to be bounded and completed before the deadline. As mentioned before, the IoT system may involve the use of a dedicated network or one with an uncertain stability. In the first case, we can compute the worst case end-to-end delay in an interaction, noted $\Delta_{i}$, from the producer to the consumer as follows:

(1) $$\Delta_{i}=C_{\mathrm{con}}+\delta_{n}+C_{\mathrm{prod}}$$

where $C_{\mathrm{con}}$ and $C_{\mathrm{prod}}$ stand for the computation time in the consumer and producer, respectively, and $\delta_{n}$ is the delay in the communication network. With it, a scheduling policy in the producer, network, and consumer can be implemented to guarantee deadlines.

If the network is not dedicated (i.e., its stability is uncertain), the situation is much more complex. Although Equation (1) is still valid, $\delta_{n}$ is not always predictable. In this case, the processing in hard real-time is not feasible as tasks may lose their deadlines while being delayed in the network. In cases where some deadlines can be lost without jeopardizing the correctness of the system (soft real-time), the delay in the network can be assumed to be the worst measured delay in the connection negotiation process and it can be updated in the next exchanges to keep the *expected delay* close to the *worst case delay* measured in the last transactions, for example, in the last 10 exchanges.

In order to deal with unknown delays in the network, we have extended the MQTT protocol [9] that, according to the analysis conducted on the related work, is closest to this proposal. The next section presents such an extension.

## 4. Modeling the Publish–Subscribe Paradigm on the SRTI Model

This section presents the implementation of the publish–subscribe interaction scheme, considering the structure of the IoT scenario and design decisions presented in the previous section. In order to do that, we specify the behavior of the processors (subscribers), terminals (publishers), and brokers (publisher/subscriber) when the SRTI model is used to support interactions in the IoT study scenario. The terminals are associated to the sensing layer in the IoT model. In this scenario, they are either producers of data (sensors) or consumers (actuators). Provided that most participants in the sensing layer are sensors, we associate terminals to publishers.

As introduced in the previous section, the processors transform data coming from sensors into information that is available for the system components. For this, the processors subscribe to topics in the different brokers. For the rest of the presentation, we assume that terminals are associated with sensors. The terminals act as actuators; therefore, they are not publishers but subscribers that receive commands to execute. Nevertheless, the proposed analysis is still valid. Similar to MQTT, the SRTI model provides a set of primitives that order the communication among processors and terminals through brokers, as illustrated in Figure 2. Typically, the smart solutions working on the IoT scenario interact directly with the processors to monitor relevant variables, to eventually deliver notifications, to make decisions, and to take particular actions when required. The processors subscribe to the services provided by the brokers (usually, information provision) given that the latter have a cooperation agreement (contract) with the terminals linked to them (i.e., sensors and actuators).

Next, we specify the behavior of the terminals, processors, and brokers using a finite state machine (FSM). We assume the connection among these components is already established; therefore, their behavior specifications are focused mainly on the exchange of data messages. In the case that a certain node operates like terminal or processor (i.e., publisher or subscriber), it may assume both operating modes, switching between them according to the role played in the system operation.

### 4.1. Terminals: Behavior Specification

**Figure 5:**
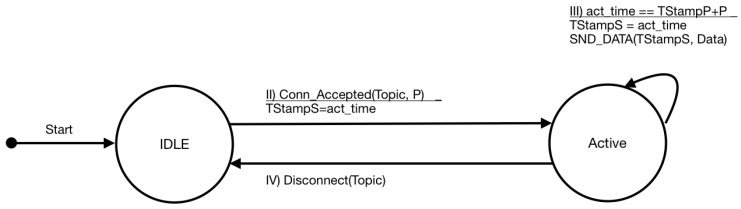
Finite state machine (FSM) representing the behavior of the terminals.

As mentioned before, the terminals correspond to the publishers in the publish–subscribe pattern. The behavior of these components is simple since, while connected to a broker, it mainly uploads data with a given periodicity. Figure 5 shows the finite state machine for the terminal. The only modification that has to be incorporated within the data is the timestamp and the period. This information is used by the broker to keep updated the delay between the publisher and the broker and to evaluate if the data will potentially arrive before the deadline to the subscriber.

### 4.2. Processor: Behavior Specification

**Figure 6:**
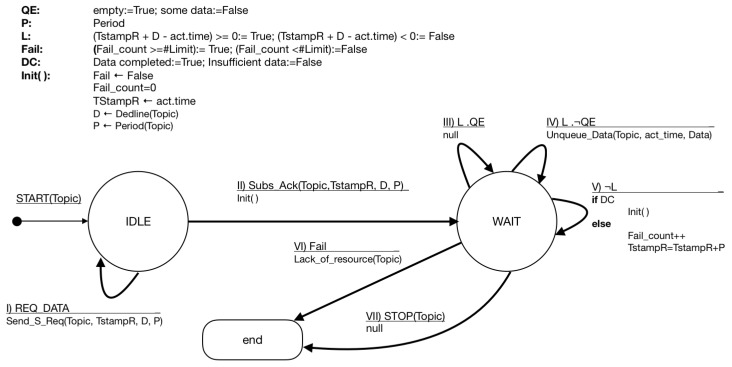
FSM representing the behavior of the processor.

In the case of the processors (i.e., the subscribers), their behavior has to deal with timestamps and deadlines. As it can be seen in Figure 6, the processor connects to the broker and remains connected while this latter forwards data received from the terminals. In the first state, the processor is IDLE waiting for an activation from the software application controlling the IoT scenario. Once the application requests data to the processor, REQ_DATA, it sends a SEND_S_REQ(Topic,TStampR,D,P) to the corresponding broker. The parameters identify the topic, the actual time of the request, the deadline, and the period. The *topic* is a variable used to identify classes of requested information (e.g., weather, traffic, or pictures of surveillance cameras). When the broker accepts the subscription, Subs_Ack(Topic,TStampS,D,P, the processors goes to the WAIT state.

When the processor is in the WAIT state, it evaluates four Boolean variables related to the subscription process.

- Queue Empty QE is true if the receiving data queue is empty, and it is false when at least one valid data has been received.
- Elapsed time *L* is true while the deadline has not been reached. It actually accounts for the available time to receive data before the deadline expiration. Once the deadline is over, this variable turns to false.
- Fail *F* is true if the amount of failures is greater than Limit. The processor records, in Fail_count, the number of times that data has not arrived within its deadline. When Fail_count≥Limit, *F* turns true.
- Data completed DC is used to identify if enough data has been received for the processing. In this case, the variable is set or reset by the application handling the queue.

The transitions associated to the WAIT are determined by the values of the Boolean variables. In the third transition, there is time (L=true) and the queue is empty; therefore, it remains in the WAIT state. The fourth transition corresponds to the case in which there is time and some data has been received. In this case, the data is unqueued and delivered to the application for its use. In the fifth transition, there is no more time and the state machine decides if it has to increment the failure count or to reset all the parameters for the next period. In this case, the DC is used to evaluate if the data received is enough for the application. When the number of failures is over Limit, it goes to the END state, indicating that there is no resource associated to the requested topic. The application may end the subscription with a STOP signal.

The services available for subscription provide single or multiple data, which is indicated using a wildcard (as in MQTT). In single data services, the answer has a single structure and semantic, but in multiple data services, that structure and semantic should be informed to the subscribers in order to allow them to properly understand the received information. Wildcards are single or multiple level. In the first case, it is used to collect the information in hierarchical topics. For example, while checking the temperature in a city, “City/+/Temperature/”, it is not relevant if the information comes from downtown or a park, whichever is available is good for that subscription. In the case of a multi-level wildcard, all the information from a specific location is relevant, for instance, “City/Downtown/#” would collect all the available topics in the downtown of a city.

### 4.3. Broker: Behavior Specification

In the publish–subscribe model, the broker is a key element that connects the actions of the publishers and subscribers. Its main purpose is to collect data from publishers and to then distribute it to the subscribers. The broker interacts with both publishers and subscribers, making its operation the most complex within the protocol. In this work, on top of the broker, the IRTA is in charge of aggregating data and producing if possible new publications to satisfy the subscribers requirements.

**Figure 7:**
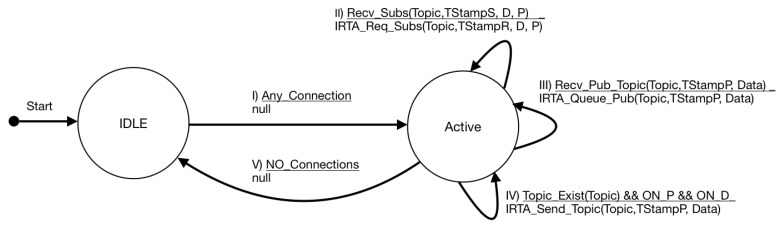
State machine diagram for broker.

Figure 7 presents the FSM for the IRTA/broker. We assume that the connection, publishing, and subscription primitives follow the standard MQTT protocol. The IRTA agent receives all the publications from the broker. When a subscriber requires a certain topic, the IRTA verifies if it has the topic in the broker directory or if it is possible to aggregate information coming from other publishers to satisfy the subscription. If part of the data is missing, it checks with other brokers to determine if it is possible to complete the necessary information. We extend this functionality by incorporating in the subscription process the identification of the period, the timestamp, and the deadline for the transmissions.

Context information (typically metadata) can also be used to determine the validity and relevance of a piece of information when the answer of a request should be created. For this purpose, it is important to have the geolocalization of the sensor, the quality of the measure, and the time at which the data has been collected as explained in Section 3. In this case, it is not necessary to know the IP address of the device.

The broker becomes Active whenever a valid connection is established to either publisher or subscriber (transition I). In the same way, it becomes Idle when there are no more connections (transition V). In transition II, the broker accepts a subscription with a certain timestamp TStampR, deadline *D*, and period *P*. With the first parameter, the broker can evaluate the remaining time it has to send the topic requested to the subscriber. The third transition describes the registration of the publisher with a certain topic and period. It also has the time at which the data was produced. In both cases, the IRTA is involved in the processing and matching of publishers and subscribers. Finally, the fourth transition describes the instant in which the broker has the topic. Provided that it is within the deadline and the period has elapsed, it must send the topic/data to the subscriber.

## 5. Implementation Details

This section describes the extensions proposed to the MQTT protocol in order to translate the SRTI model into a messaging system for application development. These extensions consider the version 3.1.1 of the protocol.

**Figure 8:**
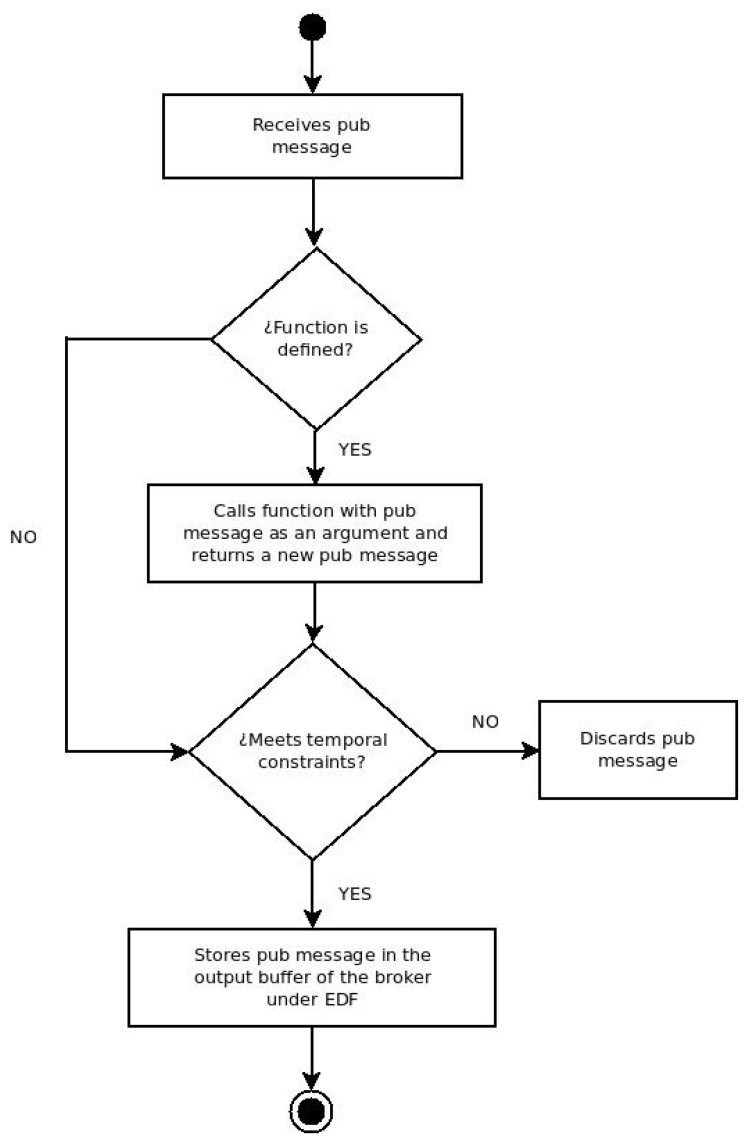
High-level activity diagram of a publication message processing of the intelligent real-time agent (IRTA).

There are two points to take into account for this work. The first one deals with how a MQTT broker should interact with the IRTA. The second one indicates how to adapt the specification of this version of MQTT, so that publishers, subscribers, and brokers can send the additional information needed by the IRTA to provide the functionalities described in previous sections. Figure 8 shows an activity diagram of the IRTA agent.

The communication between the MQTT broker and the IRTA is bidirectional; this means that both components can send and receive data. On the one hand, the broker will send information about both the subscriptions and publications that it receives. On the other hand, the IRTA will store the information of the subscriptions (e.g., the topic, timestamp, deadline, and period). Every time it receives a publication, it performs the following steps:

1. If there is a function defined by the user, it will be applied taking the publication message as an argument. The output of this function will be a new publication message that will replace the previous one. For example, the original publication could be a temperature value with the topic “Full_Temperature” with an accuracy of two bytes. However, the function applied rounds it up to one byte because that much precision is not needed; therefore, to save bandwidth, it is published under a new topic named “Shrunken_Temperature”.
2. The publication message product of step 1 is submitted to an analysis of temporal restrictions to validate if it is within the expectations of the subscriber or not. This analysis corresponds to transition VI of the state machine described in Section 4.
3. If from the previous step it is decided that the publication is valid, it is queued in the output buffer of the broker following the EDF scheduling algorithm.

### 5.1. Extensions to MQTT

Regarding the protocol specification, this work proposes three major extensions to make it suitable to allow soft real-time interactions in IoT scenarios. The first one is to add a topic registration related to the MQTT control packets, similar to MQTT-SN, which a variant of the protocol that introduces a way to register a topic in order to reduce packet sizes. The aim of the topic registration in SRTI is not to shrink the packets, but to inform the temporal data associated to the publisher and the periodicity of the topic. Therefore, its variable header should have the timestamp of the publisher, the period in which it will send the corresponding data of that topic, and the length of the topic along with its name. The packet identifier value could be 0 or 15, both currently unused.

The second extension implies modification of the headers of the PUBLISH and SUBSCRIBE MQTT Control Packets, adding in both a timestamp of the issuer. Moreover, in the case of SUBSCRIBE, also add the period and deadline in which it is expected to receive the data of that topic.

The third extension considers adding a new return code in the SUBACK package, so that the broker can reject a subscription in case it cannot offer data with the temporary parameters required by the subscriber. The value of the return code could be any between the failure code 0x80 and the maximum available 0xFF.

### 5.2. Code and Example Repository

MQTT has been implemented for personal computers with the Mosquitto [48] and Mosca [8] applications. Using the second one, we implemented the extensions to the broker that add the IRTA to such a component. In [49], the code of this implementation can be downloaded in order to install the Mosca extension. Moreover, this work also makes available an example that shows the way in which a subscriber requests a value that is obtained by processing two publications in the broker. In [50], a simple graphical interface is available to configure the Mosca application, allowing it to implement different IRTA functions that can be added by the system administrator.

## 6. Evaluation of the IRTA Extension

The proposed architecture and its implementation on Mosca was evaluated through three different experiments designed to validate the system performance; each experiment shows different aspects. In the first one, the system has two publishers: one subscriber and a broker with IRTA. The subscriber requires a topic that is not available in the broker directory, but the IRTA has a function defined that can allow the subscription using two topics coming from two publishers.

In the second experiment, the system has a publisher, a subscriber, and a broker with IRTA. The subscriber requires a topic with a certain rate that is not available within the publications. However, the topic is present with a higher rate. The broker accepts the subscription through the IRTA that subsamples the topic reducing the traffic and matching the subscriber requirements.

Finally, the third experiment involves a publisher, a subscriber, and a broker with IRTA. In this case, the subscriber requires a topic with a certain frequency. Although the topic is present in the broker, the IRTA cannot guarantee the latency and the topic is not forwarded to the subscriber reducing the traffic. The next figures show the logs obtained in each element for the different experiments.

**Figure 9:**
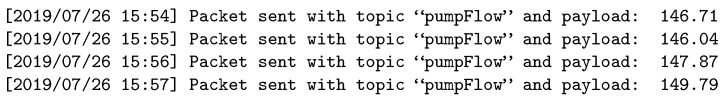
Sensor pump log.

**Figure 10:**
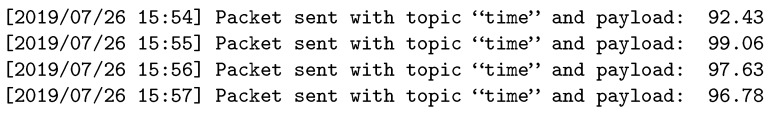
Sensor time log.

**Figure 11:**
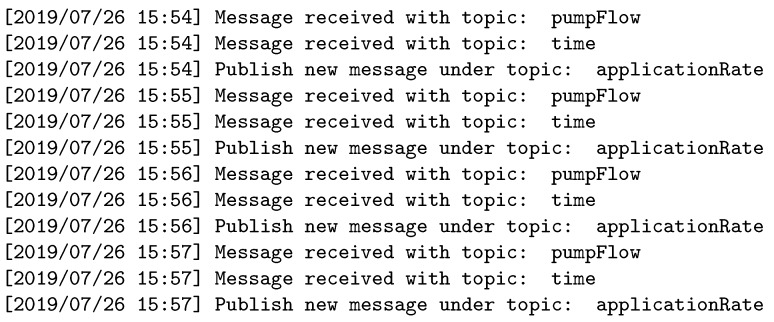
Broker log.

**Figure 12:**
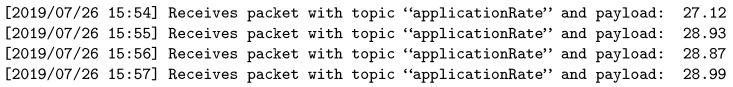
Subscriber log.

Figure 9, Figure 10, Figure 11 and Figure 12 show the log for the first experiment in the sensors, broker, and subscriber, respectively. As it can be seen, the broker waits until having the topics coming from the two publishers before forwarding the required topic to the subscriber.

**Figure 13:**
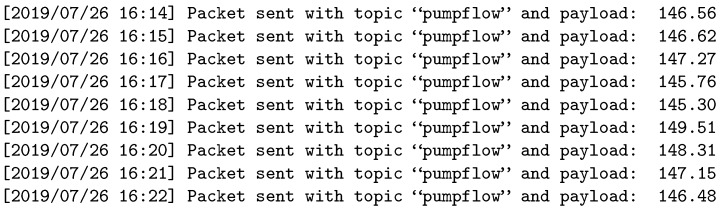
Experiment 2: Sensor log.

**Figure 14:**
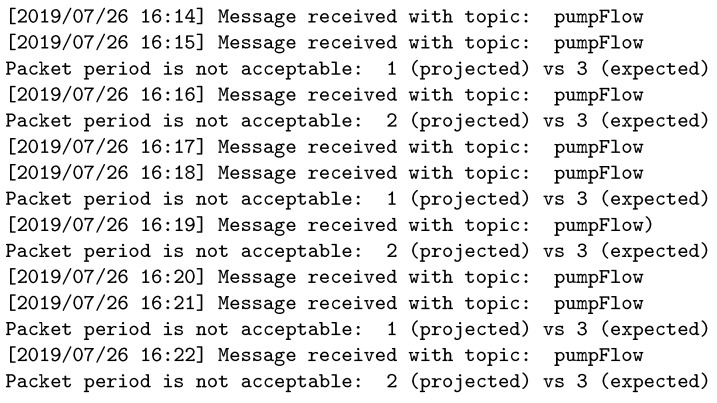
Experiment 2: Broker log.

**Figure 15:**
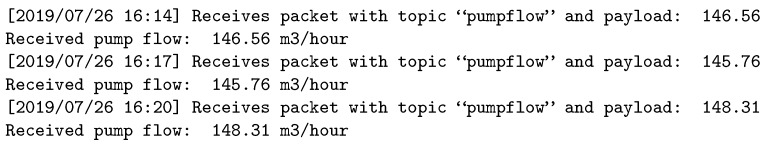
Experiment 2: Subscriber log.

Figure 13, Figure 14 and Figure 15 show the logs for the publisher, broker, and subscriber in the second experiment, respectively. Here, we can see that the publisher sends information with three times the required frequency and that the broker discards two out of three packets before forwarding the data to the subscriber.

**Figure 16:**
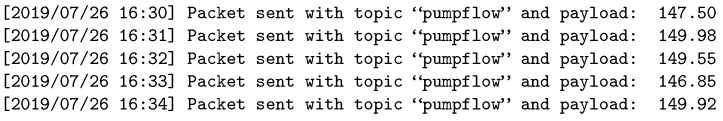
Experiment 3: Sensor log.

**Figure 17:**
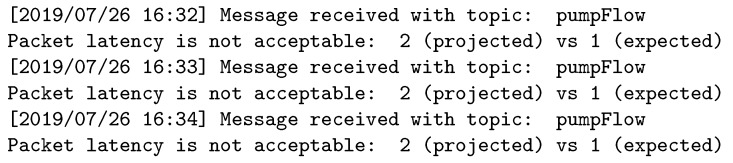
Experiment 3: Broker log.

Figure 16 and Figure 17 show the logs for the publisher and broker in the third experiment, respectively. In this case, the publisher-expected latency is higher than that the required by the subscriber; therefore, the broker discards the topic.

Table 2 presents a qualitative analysis that compares two versions of the solution required to address the three interaction scenarios described in Section 6. One version of the solution was implemented using the standard MQTT, and the other used the proposed SRTI with IRTA (SRTI-IRTA). In the three experiments, we can see that using SRTI-IRTA provides several benefits; however, we also recognize that this comparison is not fair, as such a software infrastructure implements functionalities that are not present in the current version of MQTT. Regardless of such a consideration, this comparison allows to see that SRTI-IRTA contributes to reaching soft real-time interactions in IoT scenarios and improves the implementation of the closest proposal according to the review of the state-of-the-art reported in Section 2, i.e., the MQTT protocol.

## 7. Application Scenario

This section shows an application scenario related to Precision Agriculture (PA) and describes how the proposed model can be applied to such a domain. PA is related to the use of technology to observe, measure, and decide the actions to optimize the productivity of a cultivation area, while preserving the natural resources [24,25]. As mentioned in Section 1, there are several situations that require predictable response times in PA scenarios. One typical case is the irrigation to prevent frosts and their consequences in plantations of fruit trees. When certain combinations of temperature, atmospheric pressure, and humidity are present, the frost can produce severe damage to fruit plantations, and irrigating the plants in a particular moment avoids the effects of the frost.

In PA scenarios, the synchronization among several kind of sensors distributed in the field is also required to obtain a consistent picture of the current situation. In countries where agriculture is developed in large areas, the farmers are not able to personally control the crops, not even with several employees. For this reason, it is important to count with an automatic or semiautomatic system that supervises and registers, in real-time, the different variables needed to decide which action to take.

When these irrigation systems operate autonomously, real-time interaction among the system components is required to quickly detect problems, like floods in parts of the terrain or stops of the water pumps when the plantation is in critical situations (e.g., in risk of frost). The growing of potatoes, onions, or kiwis are particularly vulnerable to these situations.

The handling of real-time constraints to improve the IoT solutions for PA is still an open issue. Although MQTT is an appropriate protocol to support the interactions among components of a PA system, it does not provides real-time guarantees for these interactions. Therefore, the extension proposed in this paper represents a way to improve the QoS of critical messages in IoT solutions that use unstable communication networks.

In the particular lots where crop is being cultivated, a set of sensors are deployed both buried within the land and measuring weather conditions in the air. For this, the temperature, ultraviolet rays and its incidence, humidity, and many others variables can be measured. These data should be sampled along the lot in different places, as lots may have several thousands of square meters. These sensors publish their data to the broker that keeps the subscribers updated. The process of retrieving the information should be made with time guarantees, since to make a decision it is important to determine the current situation of the whole lot. For example, while irrigation could be enough in one part of the lot, another part of it may still require irrigation.

Sensors deployed in the field are equipped with LoRA transceivers [29,30]. These sensors publish their measured variables to a LoRa server that implements an MQTT broker. The sensors are based on simple 8-bits microcontrollers with small RAM and ROM memories. As the proposed scenario works on open sky, they are powered with solar cells that provide energy and charge the batteries for night operation.

Typically, counting on a weather station is enough to provide the required meteorological information for a whole lot (e.g., wind direction and intensity, temperature, and humidity). This unit may provide the data independently or integrate it in a packet that includes, for instance, values of soil moisture, temperature, pH, and nitrogen. All values sampled by the sensors in the lot are used to build gradient maps on the different variables.

Other sensors can be distributed in the lot to provide information about the height of the plants and the color and state of the leaves. For this purpose, multispectral cameras can reveal information on the health of the crops. This kind of information can also be evaluated periodically with pictures taken by drones.

The scheme is repeated for each lot; therefore, there may be an important amount of information that should be processed. Lots are not necessarily close to each other; in fact, they may be several kilometers apart. The IoT systems deployed in this way facilitate the control and supervision of the lots, even when the persons in charge of them are not present in the area. Thus, the agricultural producer can make decisions based on real-time data. For instance, if there is too much moisture on certain parts of the lot but not enough on another, the farmer can decide to conduct selective irrigation.

In this kind of applications, even if deadlines are not hard, the correct operation in real-time of the system is a requirement. This aspect should be considered at the system design time. Sensors operating on batteries recharged by solar panels should consider the energy demand at the moment of sending data that has been outdated.

**Figure 18:**
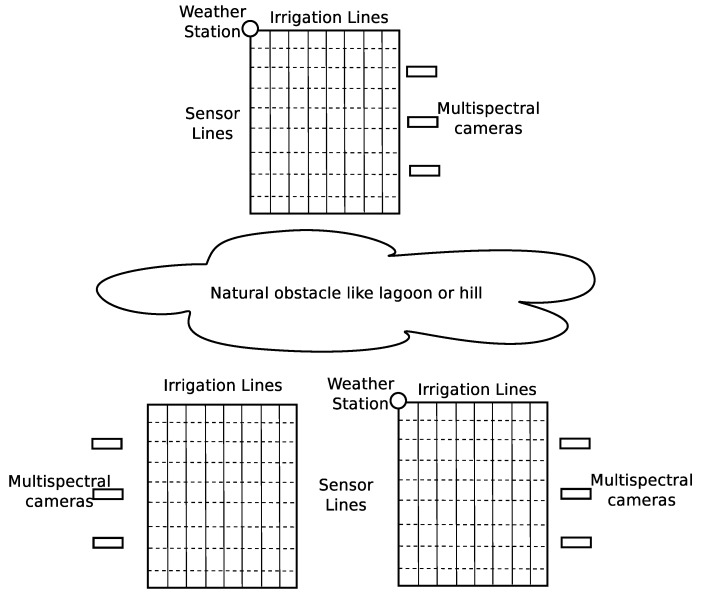
Sensors deployment in a hypothetical precision agriculture (PA) scenario.

Figure 18 illustrates a PA scenario where an IoT system is used for real-time control of the growing conditions. The sample considers three lots; each one has 800 m × 500 m. Within each lot, there are several irrigation lines that are crossed at different points by sensor lines. A multi-parametric sensor equipped with a LoRa radio transceiver is deployed in each intersection of the sensor lines. These devices can be acquired in the market at prices that vary between 10 and 30 US dollars, operate on batteries, and have a communication range of several kilometers, which make the them ideal for this kind of application. While the lot may look regular, there could be an elevation difference of 15 m between one part and another, which may lead to a zone being flooded more frequently or faster than others.

Another example that can be found in PA scenarios is having a lot with two or more types of soil. This means that the nutrients in each part and the amount of water for such areas should be managed in a different way. Figure 18 shows a schematic distribution of the sensors, which should be carefully located in order to ensure the necessary information is gathered and distributed through the system. The optimal distribution of sensors is not analyzed in this paper; therefore, it could happen that two sensors reading the same value produce different reactions in the system, as they are placed in locations with distinct requirements, even if both are within the same lot.

The transceiver of the system uses a small antenna that operates without the need to be at the site of the receptor. The weather stations may act at the same time as gateways to a non-challenged network, for instance, a mobile telephone line with 4G technology. In this case, the gateways act as MQTT brokers, and all the information coming from the sensors is collected and processed in the IRTA. Subscriptions to the brokers can be made by other gateways, processing servers, or end-users.

While the two lots in the bottom of Figure 18 may use the same weather station, the third lot has its own. With this, we remark the fact that, in the first place, the sensors may be in rather large natural scenarios in which weather conditions may vary and that, in the second place, the amount of information in the first two lots may be too much for a single broker to process in real-time. If the lot has a different shape, as the one in Figure 18, or there are not enough available sensors to cover the whole lot in a regular way, it will be crucial to analyze the structure of the lot and to install sensors in different areas, grouped by common characteristics.

The grid of the sensors shown in Figure 18 can be mapped within the lots using a matrix notation, where $s_{ijk}$ identifies the sensor within the *i*th lot, in the *j*th row, and in the *k*th column. As we assume all of them are similar within a class, each sensor in the lot will have a general name with the structure mentioned in Table 3. In the case of the weather stations, they follow the same idea. Each weather station is noted $ws_{i}$, and it has associated a lot or set of lots depending on the geography and location. Table 4 shows a possible URI assignment.

Finally, the multispectral cameras are notated as $msc_{ij}$, where *i* reflects the lot in which the camera is sampling the plants and *j* identifies the individual camera within that lot. The URI in this case is just “http://www.iotagriculture.com/mscij/spectral”, and it provides the intensities in each wavelength light. Using this sensor-naming scheme, it is possible to accomplish with the identification requirements of the MQTT protocol; particularly, it requires the identification of the topics informed by each sensor.

With the network deployed and the connection working, the user may require any node within the IoT ecosystem for appropriate information. Although we can impersonate the user, the system may work autonomously and implement some kind of decision algorithm to determine if it is necessary, for instance, to irrigate the whole lot, to irrigate part of it, or to not irrigate. The same idea can be applied to spreading herbicides or pesticides in the lot or to making any other action to maximize crop production.

## 8. Discussion

The model presented in this paper is oriented to handling soft real-time quality of service based on MQTT TCP/IP communications in the open Internet. Although there is an increasing use of sensors and end devices to provide actual information on different areas, most applications are closed and do not work in the open Internet but on dedicated networks. At the moment, the available bandwidth provided by Internet service providers is not enough to have a full mesh network of things. Several strategies are being developed to allow connecting things in a network without the intervention of people, in a true M2M fashion. Typically, these strategies are oriented to building alternative networks that let some nodes be connected to the Internet through a gateway. As mentioned before, the literature reports several systems and architectures to support interactions among IoT components, but most of these proposals show similar limitations to support real-time interactions. Some of them consider unstable networks and others involve real-time QoS, but none of them support both features simultaneously. This situation represents a stumbling block for the development of smart systems that must ensure on-time message delivery but work on networks with uncertain instability. Almost any remote monitoring system must address these two requirements simultaneously.

In this context, both LoRa and Sigfox [51] provide similar solutions to support real-time interactions. In case of South America, LoRa is leading the market and several companies have adopted the standard to provide connectivity to sensors; particularly, LoRaWAN provides an interesting connectivity pattern. In the case study analyzed in Section 7, the LoRa server acting as gateway implements at the same time the MQTT broker.

The proposed extension to MQTT provides the possibility of discriminating between messages that may comply with the time constraints from those that are not going to satisfy deadlines. At the moment, MQTT provides a way to feed subscriptions with data produced by publishers. The broker acts as a phone directory giving the data stored in the topic queue without processing to the subscriber. In this extension, the broker incorporates an agent that has the ability to classify and aggregate data to build new information. Both aspects, the real-time control and the incorporation of the intelligent agent, reduce network traffic by avoiding the transmission of outdated messages.

## 9. Conclusions and Future Work

This paper proposes the Simple Real-Time Interaction (SRTI) architectural model, which extends the interaction scheme proposed by the MQTT protocol to deal with the delivery of messages that have time constraints. The proposal was particularly designed to address IoT scenarios where the communication link is unstable or its stability is uncertain.

While IoT is emerging as the next networking paradigm, there are still several open issues related to service discovery, energy consumption, traffic congestion, and message routing that should be solved. In the meantime, as in the past, there is a race to gain the market with the appropriate technology at the physical and link layers. Among the main competitors, Sigfox and LoRa are proprietary radio systems working in the unlicensed spectrum that have already developed solutions. The fifth generation (5G) for cell phones technology has not reached yet a standard for its deployment, but it will be available during the next year [52]. In South America, at the moment, LoRa is the technology that is being adopted by most of the IoT application developers. We have implemented the MQTT extended protocol with real-time primitives on a LoRa server acting as gateway. The proposal can be used in different areas of IoT, but specially in those related to monitoring systems that require bounded response times and operate on batteries.

The SRTI model incorporates the Intelligent Real-Time Agent (IRTA) to process published data and adapts it to the subscriptions demands when possible. Such a model was implemented through a software infrastructure named SRTI-IRTA, of which the performance was evaluated in three different IoT scenarios. The results indicate that such an infrastructure helps address soft real-time message delivery in unstable networks or in those where its stability is uncertain. The capabilities of SRTI-IRTA was compared to those from a recent implementation of the MQTT protocol, showing the former outperforms the latter in the evaluation scenarios.

Finally, we have shown how this extension to MQTT can be used in precision agriculture to monitor the weather variables and chemical conditions of the soil and to control irrigation and agrochemical products used to maximize the productivity of crops. In the system proposed for this application domain, additional information is provided by spectral cameras and images obtained by drones.

The next steps in this initiative considers performing several simulations to determine the limits of this proposal. Then, we will implement various IoT applications to see that the benefits identified in the simulations are also present in the systems deployed in real scenarios. 

## Figures and Tables

**Table 1:** Metadata for temperature sensor.

Variable	Units
Temp	C, F, or K
Location Name	Lat, Long
Period	s
Precision	8/10/12/16 bits
Reliability	High/Medium/Low

**Table 2:** Comparison of solutions implemented using Message Queue Telemetry Transport (MQTT) and Software Real-Time Interaction (SRTI)-IRTA.

Experiment	Description	Solution with SRTI-IRTA	Solution with MQTT	Benefits of Using SRTI-IRTA
1	New topic based on the values of other topics.	The broker uses a function to process, create, and publish a new message under a new topic.	An extra client is needed. It should subscribe to the topics of interest, process the values received, and publish a new message under a new topic.	(1) Less traffic in the network; (2) no extra client is needed, avoiding the overhead of having another node.
2	The publisher sends a message more frequently than what the subscriber needs.	The broker filters the messages received based on the needs of the subscriber.	The broker will send all the messages received, and it will be a task of the subscriber to discard it.	(1) Less traffic in the network; (2) less processing at the subscriber.
3	The publisher latency is higher than the one required by the subscriber.	The broker filters the messages received based on the needs of the subscriber.	The broker will send all the messages received, and it will be a task of the subscriber to analyze it and discard it.	(1) Less traffic in the network; (2) less processing at the subscriber.

**Table 3:** Topics for the sensors within the loT scenario.

Variable	URI
Temperature	http://www.iotagriculture.com/sijk/temperature
pH	http://www.iotagriculture.com/sijk/pH
Humidity	http://www.iotagriculture.com/sijk/moisture
Nitrogen	http://www.iotagriculture.com/sijk/nitrogen

**Table 4:** URIs for the weather stations.

Variable	URI
Temperature	http://www.iotagricenteringculture.com/wsi/temperatute
Wind Direction	http://www.iotagriculture.com/wsi/direction
Wind Intensity	http://www.iotagriculture.com/wsi/intensity
Humidity	http://www.iotagriculture.com/wsi/humidity
Pressure	http://www.iotagriculture.com/wsi/pressure
UV factor	http://www.iotagriculture.com/wsi/uv
